# Small cell transformation of non‐small cell lung cancer under immunotherapy: Case series and literature review

**DOI:** 10.1111/1759-7714.14180

**Published:** 2021-10-07

**Authors:** Takuma Imakita, Kohei Fujita, Osamu Kanai, Misato Okamura, Masayuki Hashimoto, Koichi Nakatani, Satoru Sawai, Tadashi Mio

**Affiliations:** ^1^ Division of Respiratory Medicine, Center for Respiratory Diseases, National Hospital Organization Kyoto Medical Center Kyoto Japan; ^2^ Division of Thoracic Surgery, Center for Respiratory Diseases, National Hospital Organization Kyoto Medical Center Kyoto Japan

**Keywords:** immunotherapy, non‐small cell lung cancer, rebiopsy, small cell transformation

## Abstract

In advanced lung cancer treatment, immunotherapy provides durable responses in some patients. However, other patients experience progressive disease and the resistance mechanisms to immunotherapy have yet been fully elucidated. Small cell transformation of non‐small cell lung cancer (NSCLC) is commonly recognized as one of the resistance mechanisms to epidermal growth factor receptor (EGFR)‐tyrosine kinase inhibitors in *EGFR*‐mutant NSCLC treatment. As a resistant mechanism for immunotherapy, we report the first case of small cell transformation in 2017. Since then, eight similar cases have been reported and the concept of small cell transformation is now becoming more prevalent as a mechanism of immunotherapy resistance. In our facility, we have experienced four cases of small cell transformation after immunotherapy (including the reported case in 2017). The histology of each primary tumor was squamous cell carcinoma, large cell type neuroendocrine carcinoma, or poorly differentiated NSCLC. None had driver gene mutations. Nivolumab was administered in all four cases and atezolizumab was administered as a next line to nivolumab treatment in one case. The best response to immunotherapy was partial response or stable disease. There was a wide range of periods from the start of immunotherapy to confirmation of small cell transformation (from 2 weeks to almost 3 years). In conclusion, small cell transformation is an important resistance mechanism in cancer immunotherapy. When NSCLC progresses after immunotherapy, the possibility of small cell transformation and rebiopsy should always be encouraged, as it leads to clarification of the resistance mechanisms and frequency.

## Graphical abstract

Small cell transformation is an important resistance mechanism in cancer immunotherapy. When non‐small cell lung cancers progress after immunotherapy, we should always consider the possibility of small cell transformation and rebiopsy should be encouraged. Herein, we report four cases of tumor transformation from non‐small cell lung cancer to small cell cancer under immunotherapy.

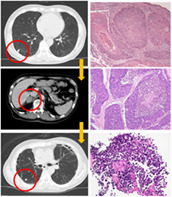

## INTRODUCTION

Lung cancer is one of the most diagnosed cancers and the leading cause of cancer death worldwide.^1^ Since 2015, immunotherapy has become a major pillar in advanced lung cancer treatment.^2^ While some patients achieve durable responses with immunotherapy,^3^ other patients experience progressive disease. However, the resistance mechanisms to immune checkpoint inhibitors (ICIs) have yet been fully elucidated. Small cell transformation of non‐small cell lung cancer (NSCLC) is commonly recognized as one of the resistance mechanisms to epidermal growth factor receptor (EGFR)‐tyrosine kinase inhibitors (TKIs) in *EGFR*‐mutant NSCLC treatment, which accounts for 3%–14% of resistant cases.^4^, ^5^, ^6^ Since we reported the first case of small cell transformation as a resistant mechanism for immunotherapy in 2017,^7^ similar cases of small cell transformation after immunotherapy have been reported^8^, ^9^, ^10^, ^11^, ^12^ and we have experienced another three cases in our facility. In cancer immunotherapy, the concept of small cell transformation is becoming more prevalent as a mechanism of immunotherapy resistance. Herein we report four cases of tumor transformation from NSCLC to small cell lung cancer (SCLC) with immunotherapy.

## CASE REPORT

### Case 1

**FIGURE 1:**
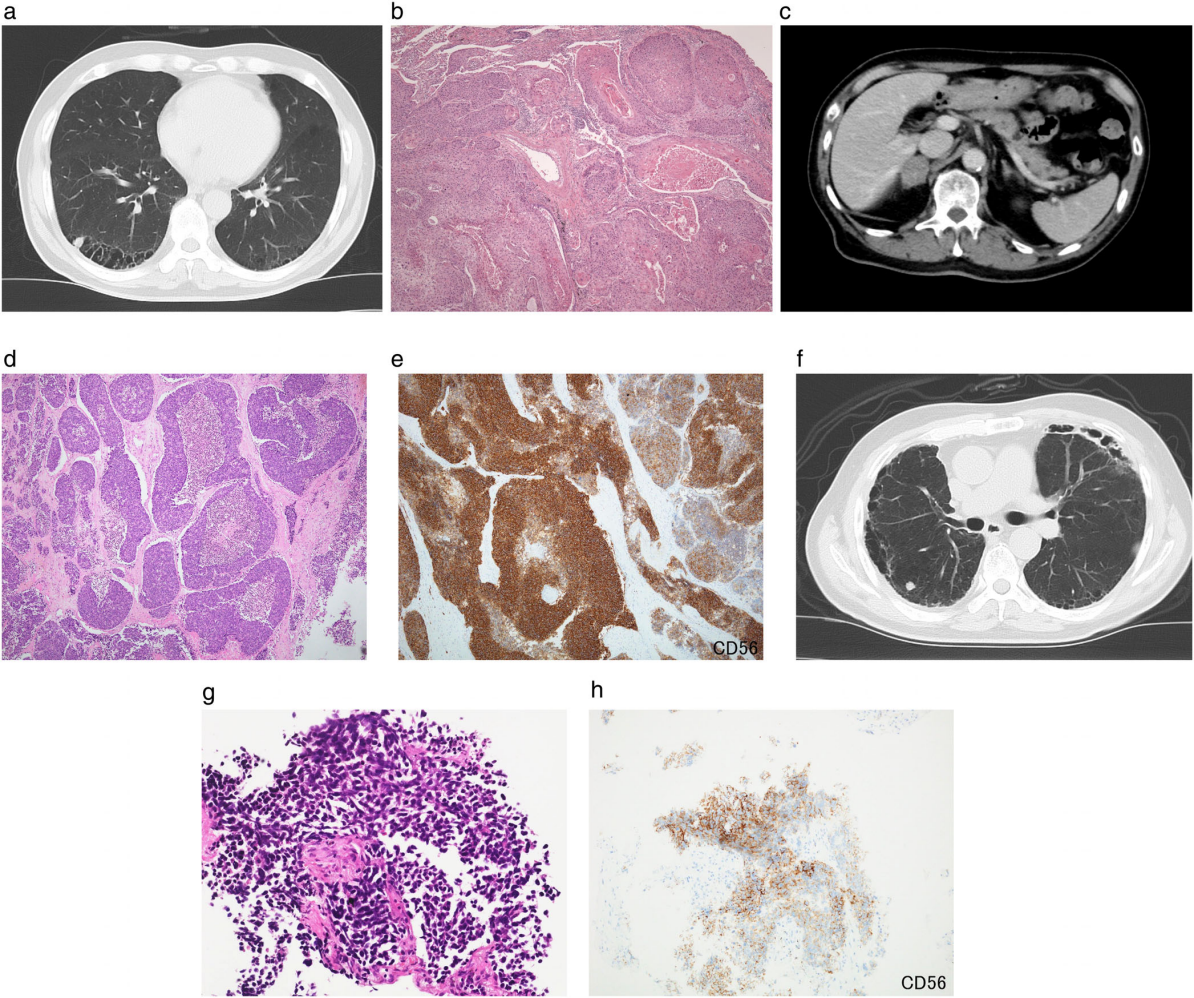
Clinical and pathological images of case 1. A computed tomography (CT) scan at first diagnosis showed a nodule in the lower lobe of the right lung (a). Lobectomy specimens showed malignant cells with anisokaryosis and hyper chromatic nuclei, which suggests moderately differentiated squamous cell carcinoma (b, hematoxylin and eosin (HE) staining ×100). After 15 cycles of nivolumab administration, a CT scan revealed progression of the lesion in the right adrenal gland (c). Specimens of right adrenectomy showed atypical and clear cells proliferating and exhibiting rosette features. Histological images of mitosis and areas of necrosis were also seen (d, HE staining ×100). Immunohistochemical (IHC) staining was positive for CD56 (e, ×100). Morphology and immunohistochemistry were compatible for neuroendocrine carcinoma, large cell type. CT‐guided needle biopsy was performed for one of the nodules in the right lung (f). The biopsy specimens showed malignant cells with scant cytoplasm and hyperchromatic nuclei, which suggested small cell cancer (g, HE staining ×400). IHC staining was positive for CD56 (h, ×400)

A 64‐year‐old man with a smoking history of 84 pack‐years was diagnosed with squamous cell carcinoma of the lung (T1aN2M0, stage IIIA) (Figure 1a,  1b). He underwent right lower lobe lung lobectomy in November 2015 followed by four cycles of adjuvant chemotherapy, a combination of carboplatin and docetaxel. Serum tumor marker levels at diagnosis were carcinoembryonic antigen (CEA) 13.5 ng/ml; cytokeratin‐19 fragment (CYFRA) 4.9 ng/ml; progastrin‐releasing peptide (pro‐GRP) 63.5 pg/ml; and neuron‐specific enolase (NSE) 10.5 ng/ml. In April 2016, a positron emission tomography (PET) scan showed multiple abnormal uptake of 18F‐2‐fluoro‐2‐deoxy‐glucose (FDG) on an occupying lesion in the liver, the right adrenal gland and a nodule in the left lung, compatible with metastases, and immunotherapy with nivolumab was started. Serum tumor marker levels at that time decreased (CEA 6.9 ng/ml and CYFRA 2.9 ng/ml). After eight cycles of nivolumab treatment, although a computed tomography (CT) scan revealed a partial response in the adrenal gland and liver metastases and stabilizing of the lung metastasis, nivolumab administration was withdrawn due to interstitial lung disease (ILD) following an immunotherapy‐related adverse event (irAE). In June 2017, a CT scan revealed progression of metastatic lesions in both the right adrenal gland and the left lung, and nivolumab was recommenced. However, after 15 cycles of nivolumab administration, his ILD relapsed in January 2018 and lung cancer treatment, including immunotherapy, was again withdrawn. In July 2018, following a CT scan, the lesion in the right adrenal gland was revealed to have progressed (Figure 1c), whereas other metastatic lesions had partially responded. In October 2018, right adrenectomy was performed and pathological findings were compatible with neuroendocrine carcinoma, large cell type (LCNEC) (Figure 1d). Immunohistochemical (IHC) staining was partially positive for CD56 (Figure 1e), chromogranin A and synaptophysin. In February 2019, a CT scan revealed progression of the metastasis in the left lung and the emergence of multiple nodules in the right lung (Figure 1f). CT‐guided needle biopsy of one of the nodules in the right lung was performed and pathology revealed small cell carcinoma (Figure 1g). There were no features of squamous cell carcinoma or LCNEC in the biopsy specimens. IHC analysis demonstrated positive staining for CD56 (Figure 1h), chromogranin A and synaptophysin. Serum tumor marker levels were CEA 5.8 ng/ml; CYFRA 2.1 ng/ml; pro‐GRP 30.7 pg/ml; and NSE 6.8 ng/ml.

### Case 2

A 74‐year‐old woman with no history of smoking was pathologically diagnosed with LCNEC (T3N0M0, stage IIB) after lung lobectomy of the left lower lobe in January 2016. Serum tumor marker levels at diagnosis were CEA 13.7 ng/ml; CYFRA 26.6 ng/ml; pro‐GRP 67.0 pg/ml and NSE 18.4 ng/ml. In August 2016, the disease relapsed in the mediastinal lymph nodes and she underwent cytotoxic chemotherapy (one cycle of carboplatin/paclitaxel followed by four cycles of vinorelbine). Owing to disease progression, immunotherapy with nivolumab was started consecutively. Although immunotherapy achieved a good partial response for the majority of the treatment period, the mediastinal lymph nodes were found to be enlarged after the 15th cycle of nivolumab administration in April 2018. Serum CEA gradually decreased from 17.2 ng/ml (when nivolumab therapy started) to 5.0 ng/ml. As a next line of treatment, atezolizumab was administered for eight cycles, which was not effective. In November 2018, endobronchial ultrasound‐guided transbronchial needle aspiration for the enlarged mediastinal lymph node was performed and small cell carcinoma was pathologically diagnosed. Serum pro‐GRP was 74.4 pg/ml and NSE was 16.5 ng/ml.

### Case 3

A 70‐year‐old man was diagnosed with squamous cell carcinoma in the left upper lobe of the lung (T4N1M0, stage IIIA) in September 2015, when serum CEA was 1.4 ng/ml. His previous medical history was notable for early‐stage esophageal cancer and he had a smoking history of 88 pack‐years. After lung lobectomy and lymph node dissection, he underwent adjuvant radiation therapy (50 Gy) for residual disease in the mediastinal surgical margin. In August 2017, CT and PET scans revealed multiple nodules in the bilateral lungs and pleura, suggesting relapsed disease. Serum CEA was elevated to 7.4 ng/ml. He underwent cytotoxic chemotherapy (carboplatin/nab‐paclitaxel) and developed febrile neutropenia during the first cycle. As his clinical course suggested no tolerability for cytotoxic chemotherapy, immunotherapy with nivolumab was chosen for subsequent treatment. Nivolumab was administered for a total of 15 cycles, including a withdrawal period because the patient developed dermatitis. The best response during immunotherapy was stable disease. In March 2019, a CT scan revealed infiltration of pleural dissemination into the right chest wall and enlargement of the mediastinal and paraaortic lymph nodes. CT‐guided needle biopsy of the pleural lesion was performed and its histology was small cell cancer. Serum CEA increased to 46.4 ng/ml and proGRP and NSE were not measured.

### Case 4

This case has already been reported.^7^ A 75‐year‐old man with a 50 pack‐year history of smoking was clinically diagnosed with poorly differentiated NSCLC, not otherwise specified (cT3N3M1a, stage IV), in February 2016. Serum tumor marker levels at diagnosis were CEA 17.5 ng/ml; Sialyl Lewis X‐i (SLX) 68.2 U/ml; pro‐GRP 299.4 pg/ml; and NSE 14.2 ng/ml. No EGFR mutations were identified, and ALK translocation was found to be negative following fluorescent immunohistochemical assay. The patient was included in a clinical trial and offered chemotherapy, a combination of docetaxel and bevacizumab, as a first‐line treatment. Following two cycles of chemotherapy, a CT scan revealed partial response and tumor markers gradually decreased (CEA 3.0 ng/ml; SLX 32.9 U/ml). In April 2016, the patient developed sigmoid colon perforation, which is a well‐known adverse event associated with bevacizumab treatment, and subsequently underwent an emergency sigmoid colon resection. Cancer treatment was withdrawn, and the disease gradually progressed. In August 2016, immunotherapy with nivolumab was initiated as a second‐line regimen. Despite three cycles of nivolumab administration, the tumor continued to progress, the right pleural effusion increased, and some subcutaneous tumors emerged. Cytopathological examination of the pleural fluid revealed SCLC. Moreover, SCLC was also identified in a biopsy from a subcutaneous tumor of the right chest, with negative staining for programmed death (PD)‐ligand 1 (L1). Serum tumor markers of SCLC were elevated (NSE 117.6 ng/ml and proGRP 5157.5 pg/ml).

## DISCUSSION

Here, we report four cases of tumor transformation from NSCLC to SCLC with immunotherapy, including the first case previously reported in 2017.^7^ We reviewed the literature and found eight reported cases, other than our cases, of small cell transformation by immunotherapy.^8^, ^9^, ^10^, ^11^, ^12^ All cases are summarized in Table 1. Almost all patients had a smoking history. The histology of the primary tumor in our cases was squamous cell carcinoma, LCNEC, and poorly differentiated NSC:C. Adenocarcinoma was not revealed in our cases, but it was revealed in four reported cases. Immune checkpoint inhibitors administered were PD‐1 inhibitors, nivolumab or pembrolizumab, apart from one case in which a PD‐L1 inhibitor, atezolizumab, was administered as a next line to nivolumab treatment (case 2). The best response to immunotherapy were various (partial response, stable disease, or progressive disease). There was a wide range in the period from the start of immunotherapy to confirmation of small cell transformation (from 2 weeks to almost 3 years). The treatment for transformed SCLC has not been established. Regimens for SCLC were chosen as the following therapy after transformation in all cases but one (continuation of nivolumab treatment).

**TABLE 1:** Characteristics of the case series and reported cases

Reference	Age at NSCLCDx and sex	Smoking history	Site of first biopsy	Histology	Immunotherapy line	ICI	Best responce	Site of rebiopsy	Histology	Common genomic profile in NSCLC and SCLC^a^	Time from ICI start to SCLC dx	Following Tx for SCLC
case 1	64, M	84 pack‐years	Lung	Sq	First	Nivo, 23 cycles	PR	Adrenal gland (op)/Lung	LCNEC/Small cell carcinoma	No mutations detected ^(a)^	29 months/33 months	CBDCA/CPT, AMR, nab‐PTX
case 2	74, F	never	Lung	LCNEC	Third/Fourth	Nivo, 15 cycles/Atezo, 8 cycles	PR/SD	LN (mediastinum)	Small cell carcinoma	No mutations detected ^(a)^	21 months	AMR
case 3	70, M	88 pack‐years	Lung	Sq	Second	Nivo, 15 cycles	SD	Chest wall	Small cell carcinoma	No mutations detected ^(a)^	16 months	ETP
Imakita, 2017^7^ (case 4)	75, M	50 pack‐years	Lung	NSCC, NOS	Second	Nivo, 3 cycles	SD	Pleural effusion/Subcutaneous tumor	Small cell carcinoma/Small cell carcinoma	No mutations detected ^(a)^	8 weeks	AMR
Abdallah, 2018^8^	65, M	35 pack‐years	Pleural effusion	Ad	Second	Nivo, 5 cycles	PD	Lung	Small cell carcinoma	‐	(Nivo, 5 cycles)	CBDCA/ETP
Abdallah, 2018^8^	68, M	‐	Right lung (op)/Left lung	Moderately diff Sq/Poorly diff NSCC	First	Pembro/CBDCA/PTX, 4 cycles followed by Pembro, 36 cycles	PR	LN (right hilar)	Small cell carcinoma	‐	2 years	CBDCA/ETP
Okeya, 2019^9^	66, M	45 pack‐years	Liver	Ad	Second	Pembro, 2 cycles	hyperPD	Pleural effusion	Small cell carcinoma	‐	5 weeks	CBDCA/ETP
Bar, 2019^10^	70, F	current	Lung	Sq with neuroendocrine features	Second/Fifth	Nivo, 3 cycles/Nivo, for 10 months	PD/SD	Adrenal gland (biopsy)/Adrenal gland (op)	Small cell carcinoma/Mixed neuroendocrine and Sq	TP53 A249S, A196T ^(b)^	16 months	Nivo (continuation)
Bar, 2019^10^	75, M	>10 pack‐years	Lung	Sq with neuroendocrine features	Second	Nivo, for 6 months	PR	Lung	Small cell carcinoma	No mutations detected ^(b)^	7 months	CBDCA/ETP
Iams, 2019^11^	67, F	50 pack‐years	Lung	Ad	Second	Nivo, 36 cycles	response	Pleural effusion/Pericardial effusion	Small cell carcinoma/Small cell carcinoma	No mutations detected ^(c)^	2 weeks	CBDCA/ETP
Iams, 2019^11^	75, F	30 pack‐years	Lung	Ad	Second	Nivo, 33 cycles	SD	LN #7	Small cell carcinoma	KRAS G12C ^(c)^	over 2 years	CBDCA/ETP
Sehgal, 2020^12^	mid‐60s, F	35 pack‐years	Lung	Poorly diff Sq	Second	Nivo, 47 cycles	PR	Lung/LN #4R, 7	Small cell carcinoma/Small cell carcinoma	TP53 R283fs*62, DKN2A R58, SOX2 amp, PIK3CA amp	21 months	CBDCA/ETP

Abbreviations: −, not described; Ad, adenocarcinoma; AMR, amrubicin; Atezo, atezolizumab; CBDCA, carboplatin; CPT, irinotecan; diff, differentiated; Dx, diagnosis; ETP, etoposide; F, female; ICI, immune checkpoint inhibitor; LCNEC, large cell type neuroendocrine carcinoma; LN, lymph node; M, male; nab‐PTX, albumin‐bound paclitaxel; Nivo, nivolumab; NOS, not otherwise specified; NSCC, non‐small cell carcinoma; NSCLC, non‐small cell lung cancer; op, operation; PD, progressive disease; Pembro, pembrolizumab; PR, partial response; PTX, paclitaxel; SD, stable disease; SCLC, small cell lung cancer; Sq, squamous cell carcinoma; Tx, treatment.

^a^ Genomic profiling by (a) oncomine Dx target test, (b) oncomine solid tumor fusion transcript kit, and (c) next‐generation sequencing.

The precise mechanism of small cell transformation with immunotherapy has not been elucidated, as well as that of EGFR‐TKI treatment. Two kinds of possible transformation mechanism are proposed, as discussed in our previous study.^7^ One hypothesis is that NSCLC cells histologically transform to small cell cancer cells. Bar et al.,^10^ Iams et al.^11^ and Sehgal et al.^12^ reported the successful detection of the same genomic features in initial and secondary tumors, which supports the histological transformation hypothesis at the cellular level. We searched gene mutations using the Oncomine Dx Target Test (Ion Torrent Personal Genome Machine Dx Sequencer; Thermo Fisher Scientific) and found no common genomic features in NSCLC and SCLC in each case. Considering the insufficient sensitivity in detecting genomic features, histological transformation in each cancer cell may be difficult to diagnose but it does exist. The other hypothesis is that the initial tumor contained both NSCLC and SCLC components, resulting in small cell predominance with immunotherapy. In some cases, neuroendocrine features were histologically revealed in pretransformation tumors. LCNEC were revealed in the primary tumor (case 2) and the secondary biopsied tumor (case 1), whereas the primary tumors had no neuroendocrine features in case 3 and 4. Bar et al.^10^ reported two cases in which the primary tumors (squamous cell carcinoma) histologically had neuroendocrine features. Because the needle biopsy or bronchoscopic lung biopsy does not reveal the whole tumor histology and the biopsied sites were inconsistent per biopsy in some cases, combined tumor at the initial examination is a possibility that needs to be addressed, but LCNEC might reflect a transition state from NSCLC to SCLC.

The frequency of small cell transformation with immunotherapy is still unknown. The main reason is that, unlike the EGFR‐TKI treatment, rebiopsy is not commonly performed after immunotherapy, which is not an exception in our facility. Nevertheless, in spite of the infrequency of rebiopsy, we experienced these four cases of small cell transformation during approximately 5 years. Sehgal et al. assumed that histological transformation with immunotherapy is under‐recognized due to the infrequency of rebiopsy.^12^

In EGFR mutant NSCLC treatment, undergoing a tumor rebiopsy is strongly recommended for the detection of secondary mutations (e.g., EGFR T790M) after disease progression with EGFR‐TKI treatment.^2^ In the era of cancer immunotherapy, the importance of rebiopsy, as well as molecular targeted therapy, should be emphasized. This can lead to the clarification of the resistance mechanisms and their frequencies.

We were unable to clarify the resistance mechanism in the cases reported here. It is important to prove the existence of a common genetic background factors to define the histological transformation hypothesis at the cellular level. We used the Oncomine Dx target test to detect common driver oncogene mutations, but none were found. Lin et al. investigated genetic profiles of combined SCLC and NSCLC and revealed a high consistency in EGFR/TP53/RB1 mutations.^13^ These mutations might be found by scrutiny with next‐generation or whole exome sequencing, but unfortunately we did not have enough specimens or budget to perform them.

In conclusion, here, we report four cases of small cell transformation from NSCLC after immunotherapy. As reported in this study, small cell transformation is an important resistance mechanism in cancer immunotherapy. When NSCLC progresses after immunotherapy, the possibility of small cell transformation and rebiopsy should always be taken into consideration.

## CONFLICT OF INTEREST

The authors report that there are no conflicts of interest.
